# Enhancement of antioxidant function in various organs in mice using combined thermal and radon inhalation treatment

**DOI:** 10.1093/jrr/rrag049

**Published:** 2026-07-13

**Authors:** Shogo Miyanaga, Ayumi Tanaka, Reiju Takenaka, Shota Naoe, Shuna Goto, Mitsuki Nagano, Kotaro Tano, Norie Kanzaki, Akihiro Sakoda, Kiyonori Yamaoka, Takahiro Kataoka

**Affiliations:** Graduate School of Health Sciences, Okayama University, 5-1 Shikata-cho 2-chome, Kita-ku, Okayama-shi, Okayama 700-8558, Japan; Graduate School of Health Sciences, Okayama University, 5-1 Shikata-cho 2-chome, Kita-ku, Okayama-shi, Okayama 700-8558, Japan; Graduate School of Health Sciences, Okayama University, 5-1 Shikata-cho 2-chome, Kita-ku, Okayama-shi, Okayama 700-8558, Japan; Ningyo-toge Environmental Engineering Center, Japan Atomic Energy Agency, 1550 Kamisaibara, Kagamino-cho, Tomata-gun, Okayama 708-0698, Japan; Graduate School of Health Sciences, Okayama University, 5-1 Shikata-cho 2-chome, Kita-ku, Okayama-shi, Okayama 700-8558, Japan; Graduate School of Health Sciences, Okayama University, 5-1 Shikata-cho 2-chome, Kita-ku, Okayama-shi, Okayama 700-8558, Japan; Graduate School of Health Sciences, Okayama University, 5-1 Shikata-cho 2-chome, Kita-ku, Okayama-shi, Okayama 700-8558, Japan; Ningyo-toge Environmental Engineering Center, Japan Atomic Energy Agency, 1550 Kamisaibara, Kagamino-cho, Tomata-gun, Okayama 708-0698, Japan; Ningyo-toge Environmental Engineering Center, Japan Atomic Energy Agency, 1550 Kamisaibara, Kagamino-cho, Tomata-gun, Okayama 708-0698, Japan; Faculty of Health Sciences, Okayama University, 5-1 Shikata-cho 2-chome, Kita-ku, Okayama-shi, Okayama 700-8558, Japan; Faculty of Health Sciences, Okayama University, 5-1 Shikata-cho 2-chome, Kita-ku, Okayama-shi, Okayama 700-8558, Japan

**Keywords:** radon, thermal treatment, antioxidant function, oxidative stress

## Abstract

Enhancing antioxidant function is a key strategy for preventing and treating oxidative stress-related diseases. Radon inhalation and thermal treatment (TT) increase the levels of antioxidant enzymes, such as superoxide dismutase (SOD). The positive effects of this combination on pain-related diseases have been reported; however, few studies have elucidated the underlying mechanisms. In this study, we examined the enhancement of antioxidant function in various organs of mice subjected to combined TT and radon inhalation. The mice were placed in an incubator once daily for 40 min at 38.5 ± 0.5°C and treated for 1, 3 and 7 days. The mice then inhaled radon at a concentration of 2000 Bq/m^3^ for 24 h. The characteristic changes in antioxidant function following combined TT and radon inhalation could be categorized into four groups: (i) increased antioxidant function in the brain and colon; (ii) increased or decreased antioxidant function, as observed in the kidney; (iii) decreased antioxidant function, such as in the lungs and (iv) no changes, such as in the small intestine, spleen, pancreas, heart, liver and stomach. Additionally, SOD may be the antioxidant enzyme most sensitive to combined TT and radon treatment. Collectively, the combined treatment may be the most effective in activating antioxidative functions in the brain and colon. This study provides new insights into the effects of combined TT and radon inhalation.

## INTRODUCTION

Reactive oxygen species (ROS) are constantly produced in the body during aerobic metabolic processes and are tightly regulated by antioxidant defense mechanisms, such as superoxide dismutase (SOD), catalase (CAT) and glutathione (GSH). However, disruption of this redox balance leads to lipid peroxidation, protein denaturation and DNA damage, thereby contributing to the onset and progression of numerous diseases, including cerebral ischemia [1], diabetes [2] and liver damage [3]. Therefore, enhancing antioxidant function is considered a key strategy for preventing and treating oxidative stress-related diseases.

Radon is a radioactive noble gas that emits α-rays. Radon therapy is typically performed in spas or radon galleries. Spa therapy alleviates symptoms of pain-related diseases, such as rheumatism [4–7] and osteoarthritis [7, 8], as well as respiratory diseases, such as bronchial asthma [9]. Because these diseases are associated with ROS, we hypothesized that radon inhalation enhances antioxidant function and previously confirmed this through animal experiments. Radon inhalation enhanced antioxidant function in various mouse organs [10], suggesting that it suppresses various diseases, including pain-related and respiratory diseases. For example, radon inhalation suppressed hippocampal neuronal damage associated with transient cerebral ischemia in gerbils [11], alcohol-induced liver damage [12] and streptozotocin-induced type I diabetes [13] by enhancing antioxidant function in the brain, liver and pancreas.

Changes in antioxidant function in rat erythrocytes following thermal treatment (TT) vary with age. For example, after seven TTs, Cu/Zn-SOD activity decreased in 1-month-old rats but increased in 6- and 12-month-old rats [14]. Heat stress in an incubator increased SOD activity in the liver and lipid peroxide levels in the kidney, whereas heat-wave exposure did not alter SOD activity in the liver or kidney [15].

Furthermore, in comparative experiments examining the effects of radon and TT in healthy humans, radon inhalation alone significantly increased levels of β-endorphin (an endogenous peptide involved in pain inhibition) and adrenocorticotropic hormone after 5 and 10 days of treatment compared with TT alone. This finding suggests that radon inhalation is effective in relieving pain [16]. Radon inhalation and TT have distinct stimulus characteristics and mechanisms of action; therefore, their combination may effectively enhance antioxidant function.

Many clinical studies have reported symptom relief with radon thermotherapy [4–9]; however, few studies have examined the enhancement of antioxidant function through the combined use of TT and radon inhalation, and the underlying mechanisms remain unclear. Therefore, this study aimed to comprehensively examine the effects of combined TT and radon inhalation on antioxidant function in mouse organs. The rationale was to provide fundamental insights into the mechanisms underlying radon spa therapy and contribute to the development of new strategies for enhancing antioxidant function.

## MATERIALS AND METHODS

### Animals

Eight-week-old male BALB/cJ mice (Jackson Laboratory, Yokohama, Japan) were used in this study. They were acclimated for 1 day under a 12-h light/dark cycle (lights on at 8:00 AM), with free access to solid feed and tap water. Each group comprised six mice. Ethical approval was obtained from the Animal Care and Use Committee of Okayama University (OKU-2024613).

### Thermal treatment

Thermal treatment conditions were determined based on a review of published literature and preliminary experiments, with parameters selected based on previous reports demonstrating enhanced antioxidant activity in rodents.

TT and non-TT procedures were conducted in an incubator (EI-300V; Az, Osaka, Japan). The incubator was preheated to 38.5°C for 1 h before treatment initiation. The mice were placed in the incubator once daily for 40 min. TT was administered for 1, 3 or 7 days. Empty mouse cages were preheated to prevent the temperature inside the incubator from dropping. If the temperature inside the incubator for the TT group reached 39.0°C, heating was temporarily halted and resumed after the temperature stabilized. The non-TT group was placed inside the incubator with the power turned off. To monitor the mice for signs of distress, the outer door of the incubator was left open, and the interior door was closed during TT. The temperature inside the incubator was recorded every 5 min in the non-TT group and every minute in the TT group. Rectal temperature was measured before and after TT using a digital thermometer (TK-60, RiXEN, New Taipei City, Taiwan). Changes and rates of change in rectal temperature over the 3- and 7-day TT periods were averaged.

### Radon inhalation

Radon inhalation was initiated within 30 min after TT, following the measurement of body temperature and body weight. The sham group underwent 24-h sham inhalation, whereas the radon inhalation group was exposed to an average concentration of 2000 Bq/m^3^ within the cage for 24 h. Immediately after radon inhalation, the animals were euthanized by excessive exposure to carbon dioxide, and the organs were collected. The target organs (brain, spleen, small intestine, liver, colon, pancreas, stomach, lung, heart and kidney) were removed and stored at −80°C until analysis.

### Biochemical assays

For SOD, CAT and total glutathione (t-GSH) assays, the samples were homogenized in 10 mM phosphate-buffered saline (pH 7.4), and the homogenates were used for the analysis. SOD and CAT activities and t-GSH content were measured as previously described [17, 18].

### Data interpretation and statistical analyses

Data are expressed as the mean ± standard error of the mean. To examine significant differences between groups, two-way analysis of variance (ANOVA) followed by Tukey’s post hoc test was performed, and differences were considered statistically significant at *P* < 0.05.

## RESULTS

### Temperature changes inside the incubator

Table 1 shows temperature changes inside the incubator. Temperature variations occurred because heating was intermittently stopped and reinitiated, as well as slight differences in incubator operation during each run.

**Table 1:** Temperature changes inside the incubator (°C)

Group	1 day	3 days	7 days
Non-TT + Sham	25.5 ± 0.15	25.5 ± 0.08	25.5 ± 0.05
Non-TT + Rn	25.6 ± 0.21	25.5 ± 0.10	25.2 ± 0.08
TT + Sham	38.0 ± 0.16	38.0 ± 0.06	38.0 ± 0.04
TT + Rn	37.7 ± 0.16	38.0 ± 0.05	37.9 ± 0.05

### Changes in the rectal temperature before and after thermal treatment

**Fig. 1:**
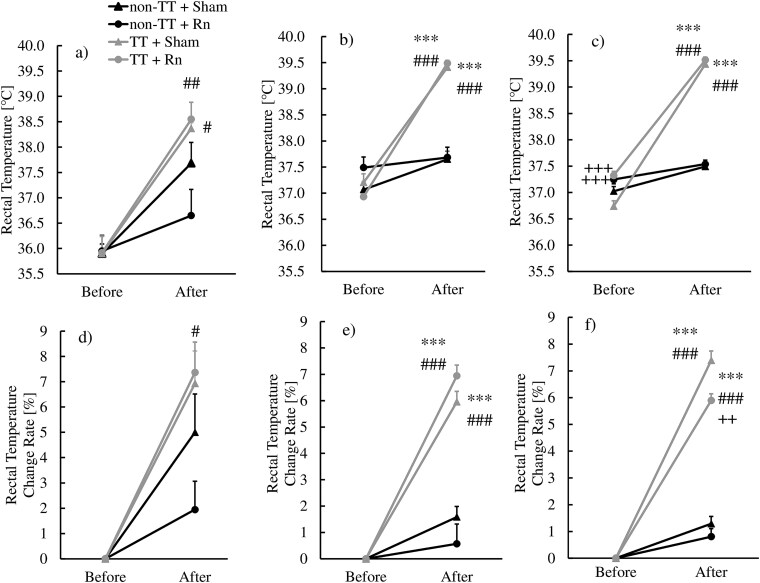
Changes in rectal temperature recorded before and after thermal treatment (TT). (a, d) day 1; (b, e) day 3 and (c, f) day 7. Mean ± standard error of the mean (SEM), *n* = 6, ^***^P < 0.001 vs. non-TT + sham; ^#^*P* < 0.05, ^##^*P* < 0.01, ^###^*P* < 0.001 vs. non-TT + radon (Rn); ^++^*P* < 0.01, ^+++^*P* < 0.001 vs. TT + Rn.

After a single TT session, the rectal temperatures in the TT + sham and TT + radon inhalation groups were significantly higher than those in the non-TT + radon inhalation group. Similarly, the rectal temperature change rates in the TT + radon inhalation group were significantly higher than those in the non-TT + radon inhalation group (Fig. 1a and d).

Among the three TT groups, rectal temperature and rectal temperature change rates were significantly higher in the TT + sham inhalation and TT + radon inhalation groups than in the non-TT + sham inhalation and non-TT + radon inhalation groups (Fig. 1b and e).

For TT, the rectal temperature and rectal temperature change rates were significantly higher in the TT + sham inhalation and TT + radon inhalation groups than in the non-TT + sham inhalation and non-TT + radon inhalation groups. Furthermore, the rate of change in rectal temperature in the TT + radon inhalation group was significantly lower than that in the TT + sham inhalation group. However, before TT, rectal temperatures in the non-TT + radon inhalation and TT + radon inhalation groups were significantly higher than those in the TT + sham inhalation group (Fig. 1c and f). These results confirm the effectiveness of TT.

### Changes in the body weight before and after thermal treatment

**Fig. 2:**
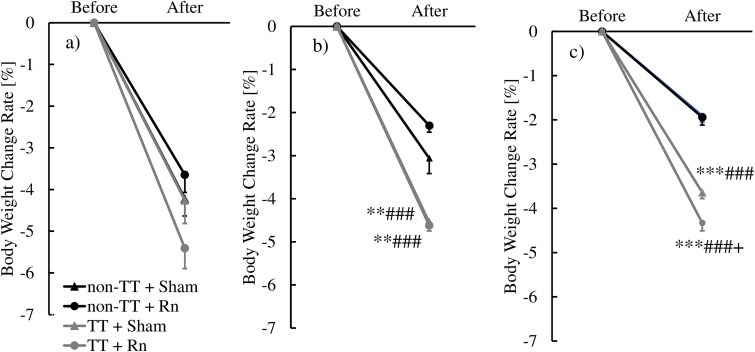
Changes in body weight before and after thermal treatment (TT). (a) 1 day; (b) 3 days and (c) 7 days. Mean ± SEM, *n* = 6, ^**^P < 0.01, ^***^P < 0.001, vs. non-TT + sham; ^###^*P* < 0.001 vs. non-TT + radon (Rn), ^+^*P* < 0.05 vs. TT + sham.

Body weight significantly decreased in the three-session TT + sham inhalation, three-session TT + radon inhalation, seven-session TT + sham inhalation and seven-session TT + radon inhalation groups (Fig. 2).

### Changes in antioxidative functions in the brain and colon after TT and radon inhalation

**Fig. 3:**
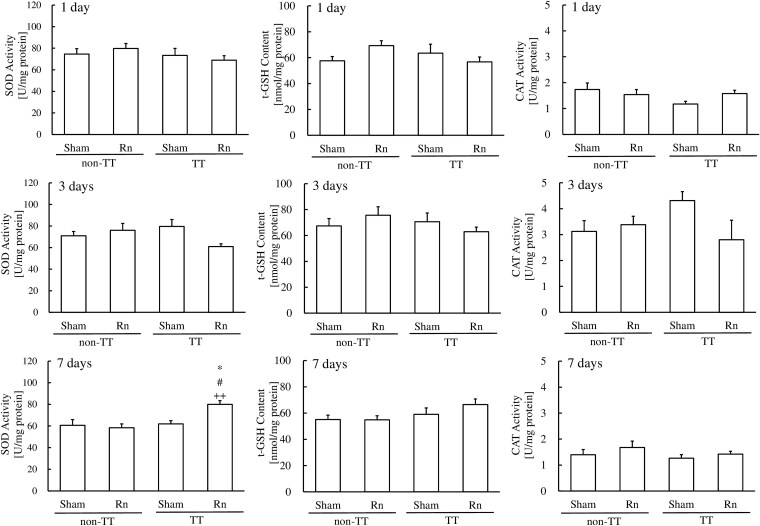
Changes in antioxidative functions in the brain after thermal treatment (TT) and radon inhalation (Rn). Mean ± SEM, *n* = 6, ^*^P < 0.05, vs. non-TT sham; ^#^*P* < 0.05 vs. non-TT + radon (Rn); ^++^*P* < 0.01 vs. TT + sham.

**Fig. 4:**
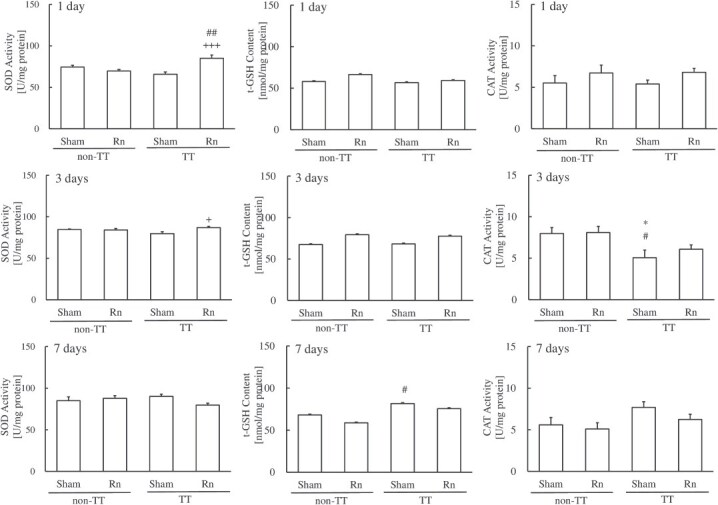
Changes in antioxidative functions in the colon after thermal treatment (TT) and radon inhalation (Rn). Mean ± SEM, *n* = 6, ^*^P < 0.05 vs. non-TT + sham; ^#^*P* < 0.05 vs. ^##^*P* < 0.01 vs. non-TT + Rn; ^+^*P* < 0.05 vs. TT + sham; ^+++^*P* < 0.001 vs. TT + sham.

In the brain, SOD activity was significantly increased in the group receiving combined 7-day TT and radon inhalation compared with that in the non-TT + sham inhalation, non-TT + radon inhalation and TT + sham inhalation groups (Fig. 3). SOD activity in the colon was significantly higher in the TT + radon inhalation group than in the non-TT + radon inhalation and TT + sham inhalation groups after a day and was significantly higher in the TT + radon inhalation group after 3 days than in the TT + sham inhalation group (Fig. 4). These findings suggest that the combination treatment activates antioxidative functions in the brain and colon.

### Changes in the antioxidative functions in the kidney after TT and radon inhalation

**Fig. 5:**
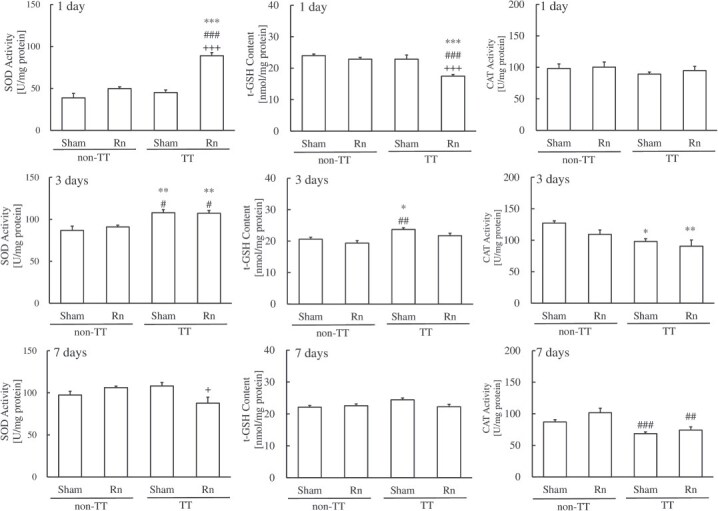
Changes in antioxidative functions in the kidney after thermal treatment (TT) and radon inhalation (Rn). Mean ± SEM, *n* = 6, ^*^P < 0.05, ^**^P < 0.01, ^***^P < 0.001 vs. non-TT + sham; ^#^*P* < 0.05, ^##^*P* < 0.01, ^###^*P* < 0.001 vs. non-TT + Rn; ^+^*P* < 0.05, ^+++^*P* < 0.001 vs. TT + sham.

In the kidneys, SOD activity was significantly higher in the single-session TT + radon inhalation group than in the non-TT + sham inhalation, non-TT + radon inhalation and TT + sham inhalation groups; however, t-GSH levels were significantly decreased. SOD activity was significantly higher in the three-session TT + radon inhalation group than in the non-TT + sham inhalation and non-TT + radon inhalation groups. CAT activity was significantly reduced in the seven-session TT + sham inhalation and seven-session TT + radon inhalation groups compared with that in the non-TT + radon inhalation group (Fig. 5). These results indicate that the effect of the combination treatment on renal antioxidant function is context-dependent, showing either increased or decreased antioxidant function depending on the experimental conditions and parameters.

### Changes in the antioxidative functions in the lung after TT and radon inhalation

**Fig. 6:**
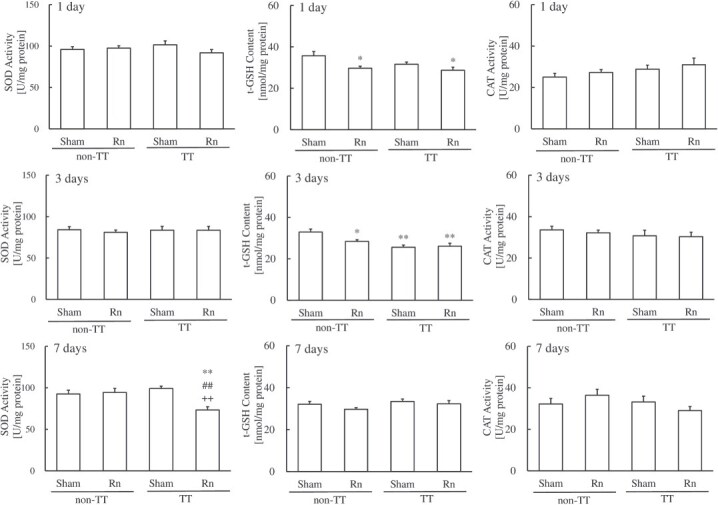
Changes in antioxidative functions in the lung after thermal treatment (TT) and radon inhalation (Rn). Mean ± SEM, *n* = 6, ^*^P < 0.05, ^**^P < 0.01 vs. non-TT + sham; ^##^*P* < 0.01 vs. non-TT + Rn; ^++^*P* < 0.01 vs. TT + sham.

In the lungs, SOD activity was significantly decreased in the group receiving combined 7-day TT and radon inhalation compared with that in the non-TT + sham inhalation, non-TT + radon inhalation and TT + sham inhalation groups. The t-GSH level was significantly reduced in the 1- and 3-day TT + radon inhalation groups compared with that in the non-TT + sham inhalation group (Fig. 6). These findings suggest that the combination treatment decreases antioxidative function in the lungs.

### Changes in the antioxidative functions in the small intestine, spleen, pancreas, heart, liver and stomach after TT and radon inhalation

The small intestine, spleen, pancreas, heart, liver and stomach showed no significant effects from the combination treatment. Although two-way ANOVA showed significant main effects, no significant interactions were observed.

**Fig. 7:**
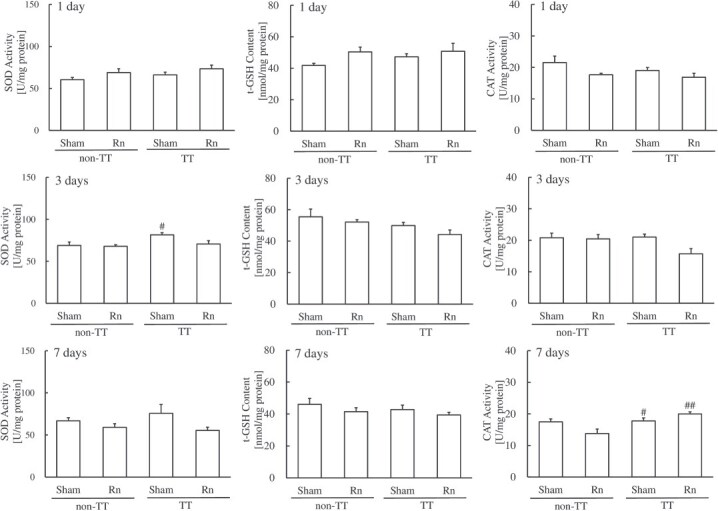
Changes in antioxidative functions in the small intestine after thermal treatment (TT) and radon inhalation (Rn). Mean ± SEM, *n* = 6, ^#^*P* < 0.05, ^##^*P* < 0.01 vs. non-TT + Rn.

**Fig. 8:**
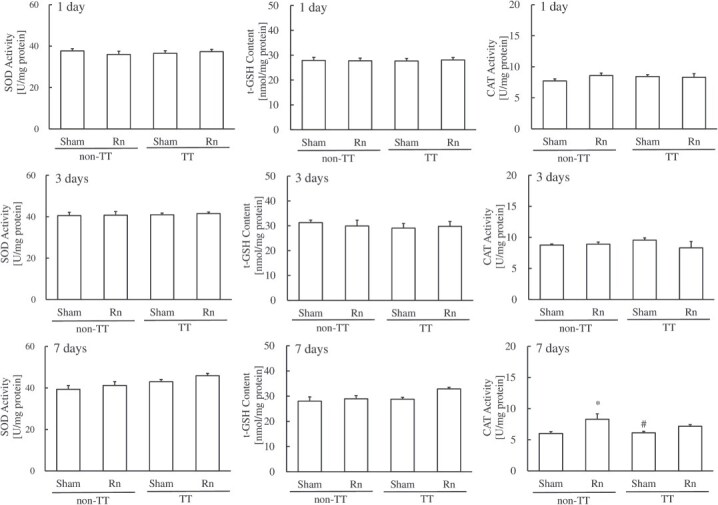
Changes in antioxidative functions in the spleen after thermal treatment (TT) and radon inhalation (Rn). Mean ± SEM, *n* = 6, ^*^P < 0.05 vs. non-TT + sham; ^#^*P* < 0.05 vs. TT + Rn.

**Fig. 9:**
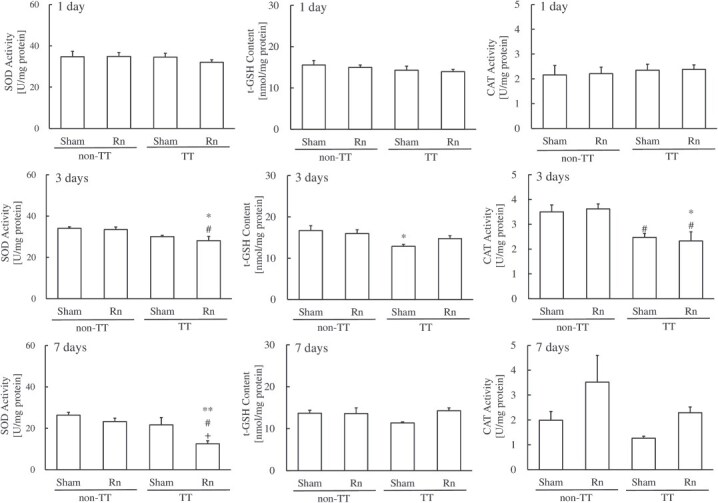
Changes in antioxidative functions in the pancreas after thermal treatment (TT) and radon inhalation (Rn). Mean ± SEM, *n* = 6, ^*^P < 0.05, ^**^P < 0.01 vs. non-TT + sham; ^#^*P* < 0.05 vs. non-TT + Rn; ^+^*P* < 0.05 vs. TT + sham.

**Fig. 10:**
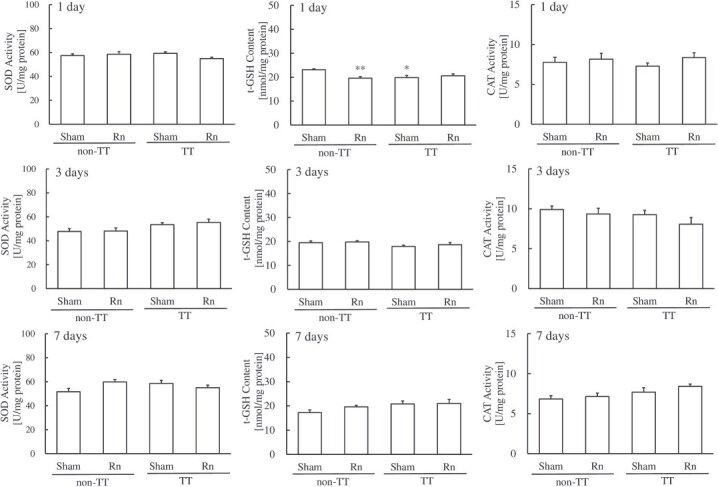
Changes in antioxidative functions in the heart after thermal treatment (TT) and radon inhalation (Rn). Mean ± SEM, *n* = 6, ^*^P < 0.05, ^**^P < 0.01 vs. non-TT + sham.

**Fig. 11:**
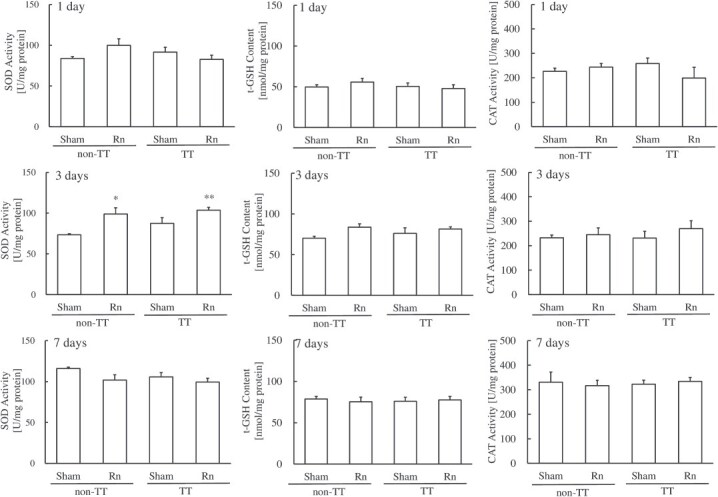
Changes in antioxidative functions in the liver after thermal treatment (TT) and radon inhalation (Rn). Mean ± SEM, *n* = 6, ^*^P < 0.05, ^**^P < 0.01 vs. non-TT + sham.

**Fig. 12:**
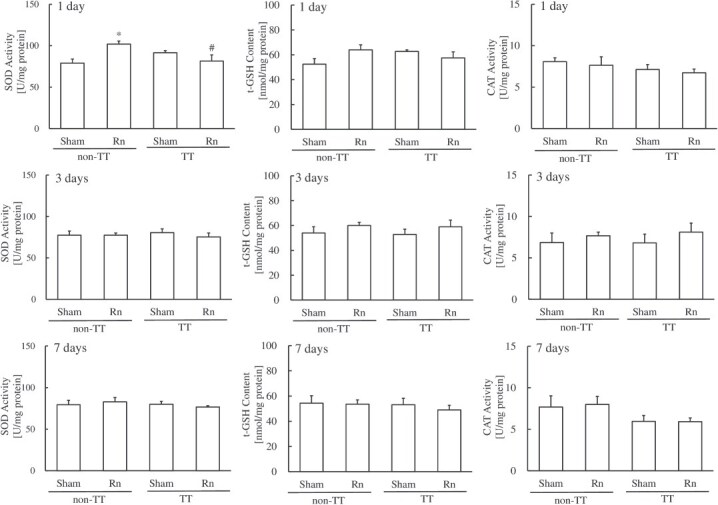
Changes in antioxidative functions in the stomach after thermal treatment (TT) and radon inhalation (Rn). Mean ± SEM, *n* = 6, ^*^P < 0.05 vs. non-TT + sham; ^#^*P* < 0.05 vs. non-TT + Rn.

In the small intestine, CAT activity was significantly higher in the 7-day TT + sham inhalation and TT + radon inhalation groups than in the non-TT + radon inhalation group (Fig. 7). In the spleen, CAT activity was significantly increased in the seven-session non-TT + radon inhalation group compared with that in the non-TT + sham inhalation group (Fig. 8). In the pancreas, SOD and CAT activities were significantly reduced in the 3-day combination treatment group compared with those in the non-TT + sham inhalation and non-TT + radon inhalation groups. After 7 days of combined treatment, SOD activity was significantly reduced compared with that in the non-TT + sham inhalation, non-TT + radon inhalation and TT + sham inhalation groups (Fig. 9). In the heart, t-GSH levels were significantly lower in the single-session non-TT + radon inhalation and TT + sham inhalation groups than in the non-TT + sham inhalation group (Fig. 10). In the liver, SOD activity was significantly higher in the non-TT + radon inhalation and TT + radon inhalation groups than in the non-TT + sham inhalation group (Fig. 11). SOD activity in the stomach was significantly lower in the combined treatment group than in the non-TT + radon inhalation group (Fig. 12). These results indicate that the effects of the combination treatment on antioxidant activity in these organs were limited and inconsistent.

## DISCUSSION

In this study, the characteristic changes in antioxidative function following combined TT and radon inhalation treatment could be categorized into four groups: (i) increased antioxidant function in the brain and colon; (ii) increased or decreased antioxidant function, such as in the kidney; (iii) decreased antioxidant function, such as in the lungs and (iv) no changes, such as in the small intestine, spleen, pancreas, heart, liver and stomach.

We previously reported that radon inhalation increased SOD activity in the brain, lung, thymus, heart, liver, stomach, pancreas, kidneys and small intestine of mice; however, this depended on radon concentration and inhalation time [10]. Another report suggested that changes in antioxidant function depended on the total antioxidant capacity of the organs [18]. This may reflect differences in oxidative stress or in the radiation dose (86 nGy to 1.4 μGy) absorbed by each organ [19, 20]. One report on the mechanisms of SOD induction revealed that radon inhalation produces ROS in the mouse brain and induces Mn-SOD, but not Cu/Zn-SOD, production [21]. This is similar to the increase in Mn-SOD induced by heat stress. Radon inhalation increased the brain nuclear factor (NF)-κB content, which regulates the induction of Mn-SOD production, in the nuclear compartment. The inhibitor of κB kinase-β, which activates NF-κB, was slightly upregulated by radon inhalation [21]. However, TT at 40°C increased nuclear factor erythroid 2-related factor 2 (Nrf2) transcription factor expression, which in turn increased the levels of the Nrf2 target proteins Mn-SOD, catalase, heme oxygenase-1, glutamate cysteine ligase and Hsp70 [22]. However, the mechanisms underlying these differences among the organs remain unknown.

Our results did not elucidate whether the combined effects were additive or synergistic. However, significant interactions were observed in the brain (SOD, 7 days), large intestine (SOD, 1 day) and kidneys (SOD, 1 or 3 days), suggesting enhanced antioxidant function. Conversely, significant decreases were observed in the lungs (t-GSH, 3 days) and kidneys (t-GSH, 1 day). These findings suggest that SOD activity tends to increase with the combined treatment, whereas t-GSH levels decrease, likely because the sulfhydryl (SH) group of glutathione is highly susceptible to oxidation.

The characteristic changes in antioxidant function following TT and radon inhalation in this study did not correspond to those we had previously reported [18]. This indicates that both TT and radon inhalation enhance antioxidant function through small amounts of ROS. However, because this study did not clarify the amount of ROS produced by the combination of TT and radon inhalation, the reason for this finding remains unclear. Given that the mechanisms underlying the enhancement of antioxidant function are similar, the effects of the combined treatment may be related to the amount of ROS produced. Based on our results, SOD may be the enzyme most sensitive to the combination of TT and radon. Additionally, the combined effects of radon inhalation and antioxidant vitamins, such as vitamins C and E, effectively inhibited alcohol-induced liver damage in mice compared with radon or antioxidant vitamins alone [23]. Our results showed findings similar to those observed in our previous study [23]. However, this study did not examine the protective effects against oxidative stress in individual organs.

The relationship between heat shock proteins (HSPs) and oxidative stress is well established [24]; therefore, HSPs were not investigated in this study. HSPs are multifunctional proteins that closely interact with antioxidants [25]. Oxidative damage to proteins and lipids contributes to HSP expression, and oxidative stress is considered a major mediator of HSP induction. In eukaryotic cells, the heat shock factor (HSF) functions as a transcriptional activator of heat shock genes, and HSF1 is activated by oxidative stress, thereby increasing the synthesis of protective HSPs [26, 27]. For example, heat stress increases hydrogen peroxide, a type of ROS, in V79 fibroblasts. A single heat shock exposure (40 min) resulted in the activation of p38 mitogen-activated protein kinase and Akt, along with increased expression of HSP70 and Mn-SOD. Similarly, during chronic heat stress, HSP70 and the antioxidant enzyme Mn-SOD were overexpressed. These inducible responses may also be caused by ROS generated by heat stress [28]. Furthermore, heat stress increases the levels of superoxide anion radicals [29, 30], hydrogen peroxide [31, 32] and hydroxyl radicals [33]. The inhibitory effects of HSPs on oxidative stress-related diseases have also been reported. For example, protective effects against ischemic cardiac injury were observed in mice overexpressing HSP70 under ischemic conditions [34–36]. This suggests that therapeutic approaches that enhance HSP70 expression may confer certain benefits [35]. Furthermore, HSPs have anti-inflammatory properties, and these effects are analogous to those of radon. These biological responses are similar to those observed following exposure to low-dose irradiation, including radon spa therapy. These differences may be due to the variations in sensitivity to TT and radon inhalation.

This study had some limitations. First, we did not investigate HSPs; therefore, we may not have accurately evaluated the effects of TT. Second, the mice were exposed to radon at a concentration of 2000 Bq/m^3^ for 24 h, which may not reflect moderate inhalation conditions. Third, the treatment methods used in this study were slightly different from those used in clinical studies. Fourth, the combined treatment produced organ-specific antioxidant responses, the nature (additive, synergistic or antagonistic) and underlying mechanisms of which remain to be determined. Additionally, the limited sample size, intentionally kept to a minimum in accordance with animal welfare principles, may have increased the risk of Type II errors due to reduced statistical power. This represents an inherent limitation of ethical animal experimentation. Finally, concurrent treatment with TT and radon inhalation was not possible due to technical constraints, which may have influenced the findings.

In conclusion, combined TT and radon inhalation treatment activated antioxidant function, particularly SOD activity, in the brain and colon. However, the effects varied depending on the organ involved. This may be due to differences in sensitivity to TT and radon inhalation. This study provides new insights into the combined effects of TT and radon inhalation. Additional research is required to characterize these combined effects in specific organs and assess their potential in mitigating oxidative stress-related diseases.
